# Regulation of Arabidopsis defense responses against *Spodoptera littoralis *by CPK-mediated calcium signaling

**DOI:** 10.1186/1471-2229-10-97

**Published:** 2010-05-26

**Authors:** Chidananda Nagamangala Kanchiswamy, Hirotaka Takahashi, Stefano Quadro, Massimo E Maffei, Simone Bossi, Cinzia Bertea, Simon Atsbaha Zebelo, Atsushi Muroi, Nobuaki Ishihama, Hirofumi Yoshioka, Wilhelm Boland, Junji Takabayashi, Yaeta Endo, Tatsuya Sawasaki, Gen-ichiro Arimura

**Affiliations:** 1 Global COE Program: Evolution and Biodiversity, Graduate School of Science, Kyoto University, Kyoto 606-8502, Japan; 2 Current Address: Department of Microbiology, Yong Loo Lin School of Medicine, National University of Singapore, Singapore 117597, Singapore; 3 Center for Ecological Research, Kyoto University, Otsu 520-2113, Japan; 4 Plant Physiology Unit, Department of Plant Biology and Innovation Centre, University of Turin, 10135 Turin, Italy; 5 Cell-free Science and Technology Research Center, Ehime University, Matsuyama 790-8577, Japan; 6 Department of Bioorganic Chemistry, Max Planck Institute for Chemical Ecology, 07745, Germany; 7 Graduate School of Bioagricultural Sciences, Nagoya University, Nagoya 464-8601, Japan

## Abstract

**Background:**

Plant Ca^2+ ^signals are involved in a wide array of intracellular signaling pathways after pest invasion. Ca^2+^-binding sensory proteins such as Ca^2+^-dependent protein kinases (CPKs) have been predicted to mediate the signaling following Ca^2+ ^influx after insect herbivory. However, until now this prediction was not testable.

**Results:**

To investigate the roles CPKs play in a herbivore response-signaling pathway, we screened the characteristics of Arabidopsis CPK mutants damaged by a feeding generalist herbivore, *Spodoptera littoralis*. Following insect attack, the *cpk3 *and *cpk13 *mutants showed lower transcript levels of plant defensin gene *PDF1.2 *compared to wild-type plants. The CPK cascade was not directly linked to the herbivory-induced signaling pathways that were mediated by defense-related phytohormones such as jasmonic acid and ethylene. CPK3 was also suggested to be involved in a negative feedback regulation of the cytosolic Ca^2+ ^levels after herbivory and wounding damage. *In vitro *kinase assays of CPK3 protein with a suite of substrates demonstrated that the protein phosphorylates transcription factors (including ERF1, HsfB2a and CZF1/ZFAR1) in the presence of Ca^2+^. CPK13 strongly phosphorylated only HsfB2a, irrespective of the presence of Ca^2+^. Furthermore, *in vivo *agroinfiltration assays showed that CPK3-or CPK13-derived phosphorylation of a heat shock factor (HsfB2a) promotes *PDF1.2 *transcriptional activation in the defense response.

**Conclusions:**

These results reveal the involvement of two Arabidopsis CPKs (CPK3 and CPK13) in the herbivory-induced signaling network via HsfB2a-mediated regulation of the defense-related transcriptional machinery. This cascade is not involved in the phytohormone-related signaling pathways, but rather directly impacts transcription factors for defense responses.

## Background

One of the significant factors determining successful plant growth and reproduction is an efficient defense against insect attacks. After herbivore feeding there is a dramatic Ca^2+ ^influx limited to a few cell layers lining the damage zone [1,2]. Signals induced rapidly by herbivore attack have been found to spread over the leaf, leading to a strong Ca^2+^-dependent transmembrane potential (*V*m) depolarization in the damage zone followed by a transient *V*m hyperpolarization in the close vicinity and a constant depolarization at distances greater than 6-7 mm [1]. These initial cues are transmitted within the plant by signal transduction pathways that include phosphorylation cascades, such as mitogen-activated protein (MAP) kinases, and the jasmonic acid (JA) pathway, which play a central and conserved role in promoting resistance to a broad spectrum of insects [3]. However, there is a missing link to downstream signaling and gene regulation for defense responses. In this network, Ca^2+^-binding sensory proteins are of particular interest, since following Ca^2+ ^influx the sensory proteins may secondarily decode information contained in the temporal and spatial patterns of the signal trafficking to control metabolism and gene expression [4].

Plants possess several classes of Ca^2+^-binding sensory proteins, including calmodulins, calmodulin-like proteins, calcineurin B-like proteins, and Ca^2+^-dependent protein kinases (CPKs) [4]. The CPKs are of special interest, since they represent a novel class of Ca^2+ ^sensors, having both a protein kinase domain and a calmodulin-like domain (including an EF-hand calcium-binding site) in a single polypeptide [5,6]. CPKs constitute a large family of serine/threonine protein kinases that are broadly distributed in the plant kingdom. For example, the Arabidopsis genome is predicted to have 34 different CPKs [7]. Arabidopsis CPK1 was the first CPK to be characterized, and is known to be activated by phospholipids and 14-3-3 proteins, which are small, highly conserved eukaryotic proteins that regulate multiple cellular enzymes, including protein kinases [8]. AtCPKs 3, 4, 6, 11 and 32 act as abscisic acid (ABA) signaling components, and are involved in ABA-responsive gene expression, seed germination, seedling growth, and stomatal movement [9-11]. Especially, AtCPKs 4, 11 and 32 are likely to interact with ABA-related leucine zipper class transcription factors [10,11], indicating the proximate involvement of CPKs in transcriptional regulation.

Curiously, little attention has been given to the role of CPKs in defense responses. Only one case has been reported: in tobacco, NtCDPK2 modulates the activation of stress-induced MAP kinases, and this interaction requires the synthesis and perception of wound hormones [12]. The role of CPKs involved in the defense response against insect herbivory has never been reported. In this report, we show the involvement of two Arabidopsis CPKs (CPK3 and CPK13) in the herbivory-induced signaling network via post-translational regulation of the defense-related transcriptional machinery. Implications for possible signal trafficking via CPKs are discussed.

## Results

### *cpk3 *and *cpk13 *mutants showed decreased transcript levels of defense genes in *S. littoralis*-damaged leaves

**Figure 1:**
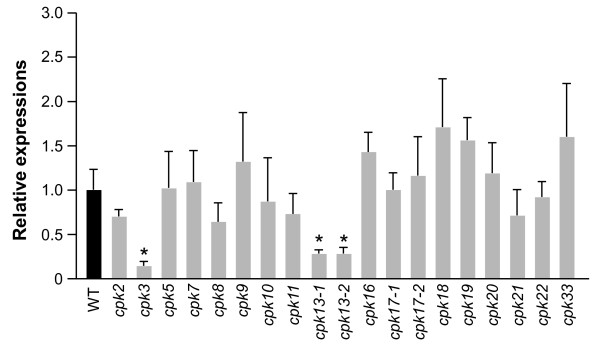
**Comparison of the transcript levels of plant defensin gene *PDF1.2 *between *S. littoralis*-infested leaves of WT and CPK T-DNA insertion mutants**. Transcript levels of *PDF1.2 *in mutant leaves (gray columns) were normalized by those of *ACT1 *measured in the same samples and expressed relative to the normalized transcript levels in leaves of infested WT plants (filled columns). An asterisk (*) indicates that mutants were significantly different from WT leaves (*P *< 0.05). WT, wild-type; *cpk2*, Salk_131765; *cpk3*, Salk_022862; *cpk5*, Salk_138808; *cpk7*, Salk_035601; *cpk8*, Salk_036581; *cpk9*, Salk_034324; *cpk10*, Salk_105108; *cpk11*, Salk_023086; *cpk13-1*, Salk_057893; *cpk13-2*, Salk_135795; *cpk16*, Salk_020716; *cpk17-1*, Salk_140527; *cpk17-2*, Salk_057146; *cpk18*, Salk_061352; *cpk19*, Salk_057587; *cpk20*, Salk_073448; *cpk21*, Salk_029412; *cpk22*, Salk_125850; *cpk33*, Salk_059467

**Figure 2:**
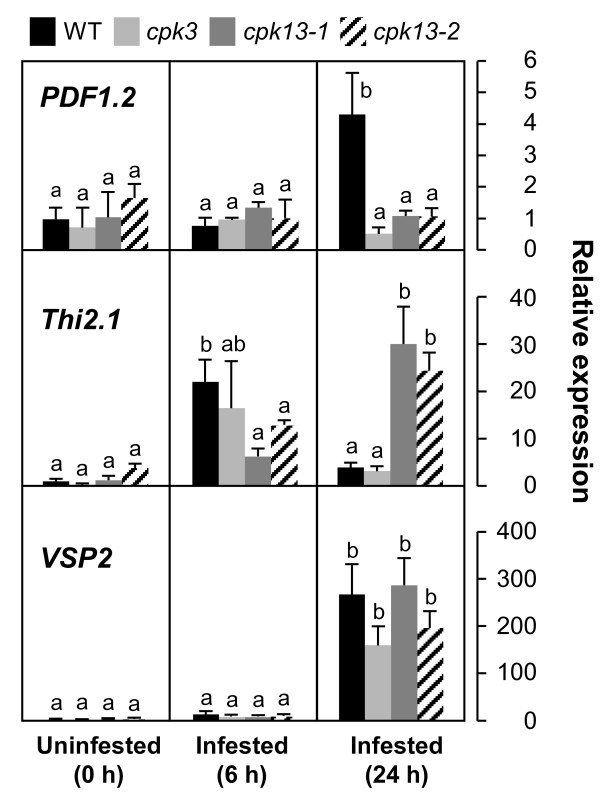
**Relative mRNA levels of defense-related genes *PDF1.2*, *Thi2.1 *and *VSP2 *in *S. littoralis*-infested leaves**. Transcript levels of genes in WT, *cpk3*, *cpk13-1 *and *cpk13-2 *leaves were normalized by those of *ACT1 *measured in the samples and expressed relative to the normalized transcript levels in the leaves of uninfested WT plants. Means followed by different small letters are significantly different (*P *< 0.05)

We obtained 19 T-DNA insertion mutant lines that were putative AtCPK-deficient mutants from the European Arabidopsis Stock Centre. Homozygous T-DNA insertion lines corresponding to each CPK gene were confirmed using the PCR method (data not shown) and challenged with herbivore damage. Transcript levels of plant defensin gene *PDF1.2*, which is induced in wild-type (WT) leaves exposed to *Spodoptera littoralis *larvae for 24 h, were investigated in the mutant leaves (Figure 1). Compared to *S. littoralis*-damaged WT leaves, T-DNA insertion line *cpk3 *(Salk_022862) and two different *cpk13 *lines (*cpk13-1 *(Salk_057893) and *cpk13-2 *(Salk_135795)) showed significantly lower transcript levels in *S. littoralis*-damaged leaves. The molecular analysis of CPK T-DNA insertion mutants and gene expression profiling of the mutants are shown in Additional file 1. In addition to our study of *PDF1.2*, the time-courses of the transcript levels of two other defense-related genes (*Thi2.1 *and *VSP2*) after herbivore attack were examined in *cpk3*, *cpk13-1*, and *cpk13-2 *leaves and compared to those in WT leaves (Figure 2). The transcript level of *Thi2.1 *in WT leaves was increased about 22 fold after 6 h but declined after 24 h. The *cpk3 *mutant showed a comparable level of the *Thi2.1 *expression during the time course, whereas the transcript levels in *cpk13-1 *and *cpk13-2 *leaves remained unchanged at 6 h, but increased after 24 h. Thus, WT and *cpk13 *probably have different temporal regulation. Throughout the time-course, the induced transcript levels of *VSP2 *were comparable between the WT and all the mutants (Figure 2).

### Possible involvement of phytohormone signaling in the herbivory-related CPK cascades

**Figure 3:**
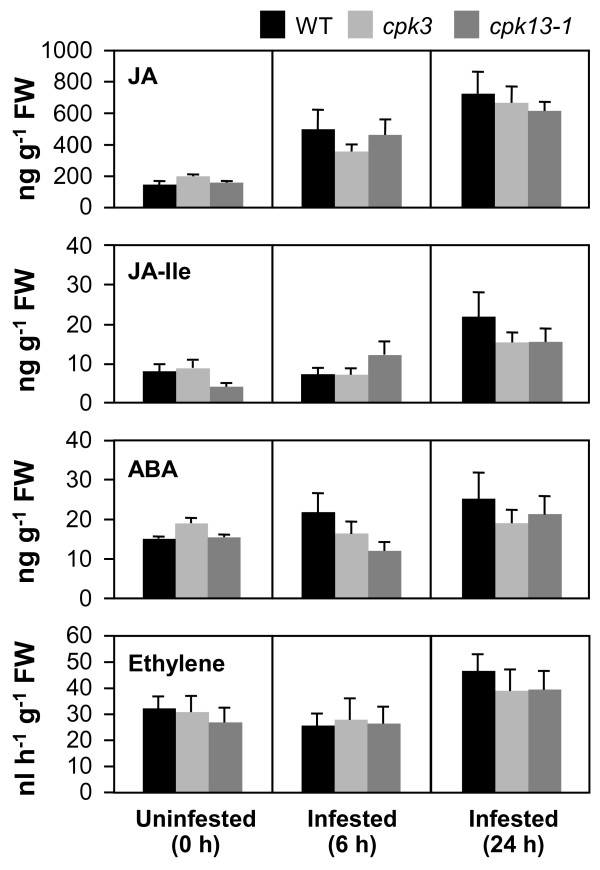
**Levels of JA, JA-Ile, ABA and ethylene in WT, *cpk3*, and *cpk13-1 *leaves fed on by *S*. *littoralis *larvae for 6 and 24 h**.

**Figure 4:**
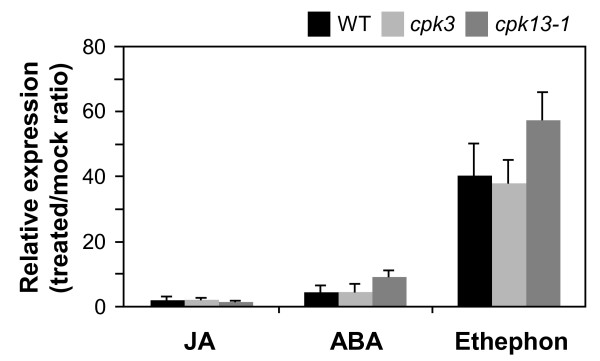
**Effects of loss of CPK function on signaling pathways for defense response**. Effect of exogenous application of JA, ABA or ethephon (an ethylene releasing chemical) on *PDF1.2 *expression levels after 6 h was investigated. Transcript levels of *PDF1.2 *in WT, *cpk3*, and *cpk13-1 *leaves were expressed relative to the normalized transcript levels in leaves of mock-treated (water-treated or buffer-treated) wild-type plants. Significant differences between treatments were not observed

To assess whether loss of CPK function affects the signal transduction involved in the defense response, we explored the biosynthesis of JA, jasmonyl-L-isoleucine (JA-Ile, an active form of JA [13]), ethylene, and abscisic acid (ABA, known to be involved in protective wound-healing processes [14]). As shown in Figure 3, all the phytohormones examined in this study were formed and accumulated at a similar rate in the infested WT, compared to the *cpk *mutant leaves (Figure 3), indicating that CPK3 and CPK13 are not upstream signal kinases for the biosynthesis of JA, ABA and ethylene. We also investigated the *PDF1.2 *expression levels in WT and in *cpk3 *and *cpk13 *mutants by applying an exogenous solution of either JA, ethephon (a chemical which releases ethylene), or ABA (Figure 4). Compared to the control, after 6 h the transcript levels of *PDF1.2 *were very slightly induced by JA or ABA to levels comparable to those in WT and mutant leaves. Ethephon treatment resulted in drastic but comparable increases of the transcript in both WT and mutant leaves. We therefore conclude that the CPK3 and CPK13 cascades are not closely linked to the above phytohormone signaling and biosynthesis cascades.

### Increased intracellular Ca^2+ ^levels in *cpk3 *leaf cells after herbivore and mechanical damage

**Figure 5:**
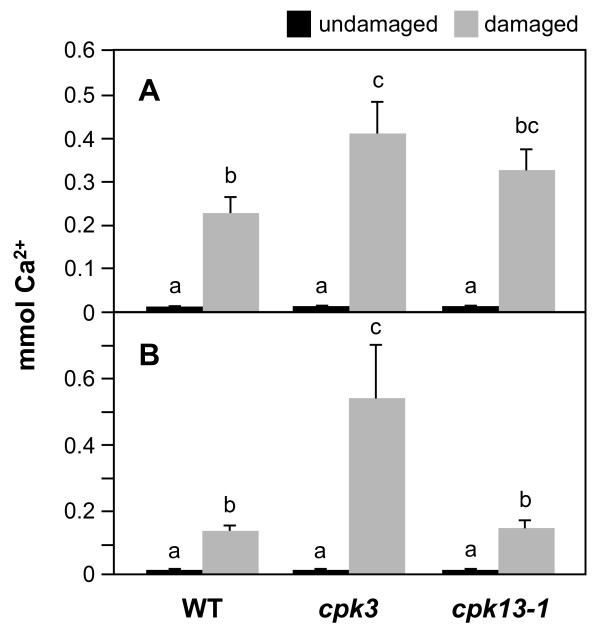
**Intracellular Ca^2+ ^levels in WT and *cpk *leaf cells**. Undamaged WT leaves served as a control. Leaves were treated with Fluo-3 AM for 1 h and damaged with a *S. littoralis *larva (A) or mechanical wounding (B). Thirty minutes after damage, the cytosolic Ca^2+ ^concentrations in leaf cells were determined and taken as the saturation value. Undamaged WT leaves served as a control. Means followed by different small letters are significantly different (*P *< 0.05).

It is interesting to note that the *cpk3 *mutant showed abnormal changes of the cytosolic Ca^2+ ^level after insect damage. As shown in Figure 5, when the membrane-permeable Fluo-3 AM [Ca^2+^-sensitive fluorescent probe] was applied to WT leaf tissues, it showed a cytoplasmic subcellular localization at sites damaged by *S. littoralis *(Figure 5A) or after mechanical wounding (Figure 5B). *cpk3 *but not *cpk13-1 *plants showed a more marked increase of the cytosolic Ca^2+ ^level after the damage, compared to WT.

### Substrate targeting of CPK3 and CPK13

**Figure 6:**
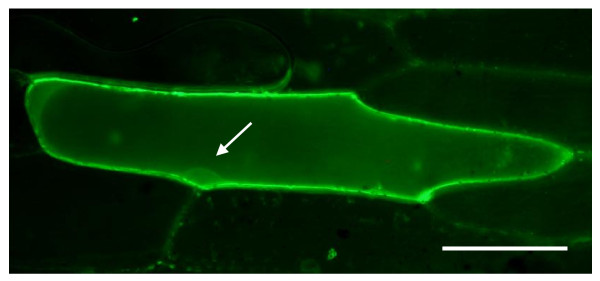
**Transient expression of the CPK13-GFP fusion proteins**. The recombinant plasmid was transformed into onion peels by particle bombardment. Cells were observed under a fluorescence microscope. Bars represent 100 μm. Arrows point to the nucleus

**Figure 7:**
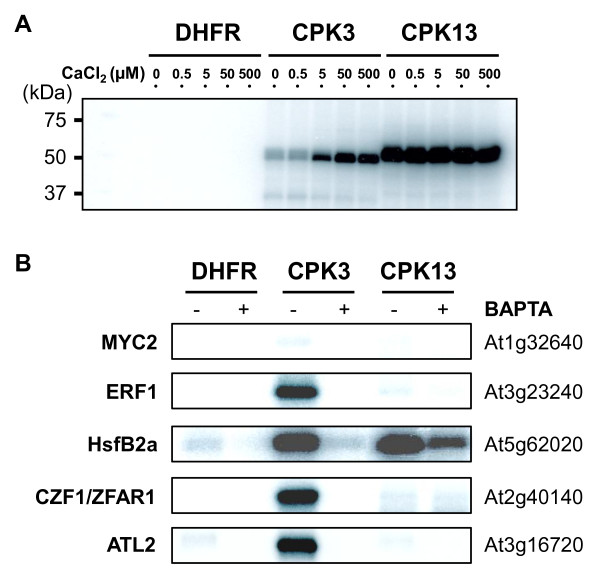
**Substrate targeting of CPK3 and CPK13**. A. Auto-phosphorylation signals from each CPK protein. Recombinant CPK was pre-treated with BAPTA (2.5 mM; a calcium chelator) and then subjected to auto-phosphorylation assays with several concentrations of CaCl_2_. B. *In vitro *kinase assays of the CPK3 and CPK13 proteins with a suite of TFs and ATL2 (following cell-free protein synthesis and purification) revealed their substrate targets. In the presence (+) or absence (-) of BAPTA (500 μM), kinase assays were performed in the presence of CaCl_2 _(100 μM). The DHFR protein served as a control. Each experiment was performed two or three times, with similar results each time. See the quantitative values in Additional file 3.

In previous studies, CPK3 was found to be localized in the nucleus and the cytosol [15]. In this study, we additionally tested the subcellular localization of CPK13-GFP fusion proteins in transiently expressing onion peels, which revealed nuclear, cytosolic and plasma membrane localizations of the fusion proteins (Figure 6). Accordingly, in order to screen the protein target of CPK3 and CPK13, protein kinase assays with 100 nuclear and cytosolic protein substrates synthesized using the wheat germ cell-free system (see Additional file 2) were carried out. CPK3 or CPK13 proteins purified from a cell-free extract were incubated with radiolabeled ATP, CaCl_2 _and the 100 distinct substrates. Both CPK3 and CPK13 showed auto-phosphorylation in the presence of radiolabeled ATP and CaCl_2 _(Figure 7A). Notably, CPK3 was auto-phosphorylated along with increased concentration of Ca^2+^, whereas CPK13 was not, indicating that CPK3 was strictly Ca^2+^-dependent. Auto-phosphorylation of CPK stringently reflects the intensity of the phosphorylation of substrate targets [16]. As shown in Figure 7B and Additional file 3, CPK3 phosphorylated three transcription factors (TFs) [JA/ethylene-inducible APE/ERF domain transcription factor 1 (ERF1) [17], heat shock factor HsfB2a (Hsf22), and the wound-inducible CZF1/ZFAR1 transcription factor [18]]. ATL2, a member of a multigene family of highly related RING-H2 zinc finger proteins that function as E3 ubiquitin ligases [19,20] and a potent regulator of *PDF1.2 *transcription [21], was also phosphorylated by CPK3 (discussed in Additional file 4). Addition of BAPTA, a calcium chelator, to the reaction mixture abolished the phosphorylation by CPK3, suggesting that these phosphorylations were strictly Ca^2+^-dependent. This result was in line with the Ca^2+^-dependent auto-phosphorylation of CPK3, described above. In contrast, the CPK13-derived protein labeling (auto-phosphorylation) was scarcely activated by the addition of Ca^2+ ^ion (Figure 7A), suggesting that CPK13 has very high sensitivity for autophosphorylation, like soybean CDPKα [22]. CPK13 strongly phosphorylated HsfB2a, irrespective of the presence of BAPTA.

Although it has been claimed that MYC2 (At1g32640) is also involved in *PDF1.2 *regulation [23], this transcription factor was not phosphorylated by either CPK3 or CPK13 (Figure 7B).

### HsfB2a takes part in the regulation of the herbivore-induced transcription of *PDF1.2*

**Figure 8:**
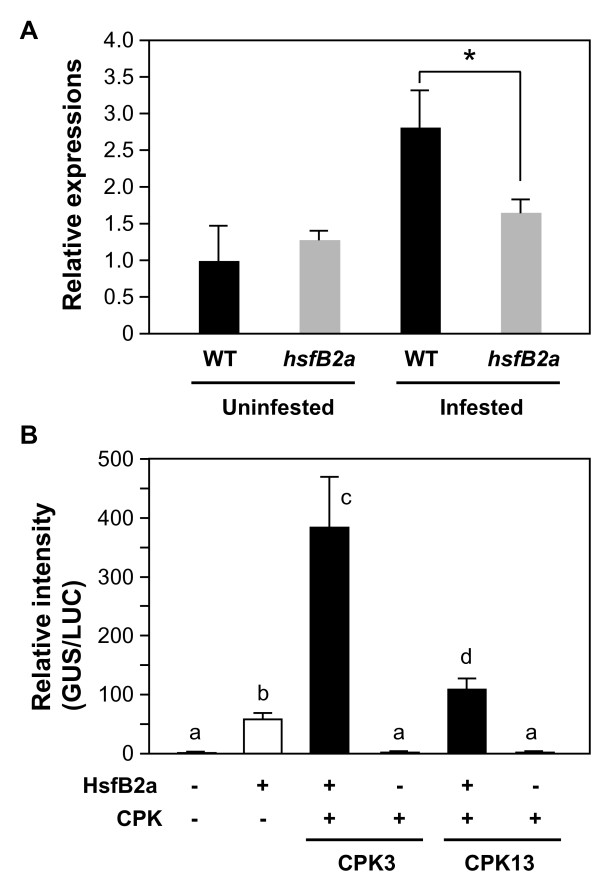
**Involvement of CPK3, CPK13 and HsfB2a in herbivore-induced *PDF1.2 *regulation**. A. Transcript levels of *PDF1.2 *in WT and *hsfB2a *mutant Arabidopsis leaves fed on by *S*. *littoralis *larvae for 24 h. An asterisk (*) indicates that mutants were significantly different from WT (*P *< 0.05). B. *PDF1.2 *promoter activity in *N. benthamiana *leaves co-expressing CPK and HsfB2a. The 1-kb *PDF1.2 *promoter region was fused to a GUS reporter gene including the intron. Transient activation of the reporter gene according to co-expressed effector(s), HsfB2a, or truncated variant of CPK3 or CPK13, in *N. benthamiana *leaves was assessed. *Agrobacterium*, carrying 35S promoter::LUC including intron, was used to normalize for the efficiency of agroinfiltration. Means followed by different small letters are significantly different (*P *< 0.05).

We investigated the *PDF1.2 *expression levels in *S. littoralis*-damaged leaves of Arabidopsis WT and HsfB2a T-DNA insertion mutants (Salk_027578) (Figure 8A). Compared to *S. littoralis*-damaged WT leaves, *hsfB2a *plants showed significantly lower transcript levels in *S. littoralis*-damaged leaves. Thus, HsfB2a appeared to be a positive regulator of herbivore-induced *PDF1.2 *expression. In addition, to investigate the *in vivo *function of CPKs, a constitutively active form of CPK and HsfB2a were co-expressed as cofactors for the transient expression of a reporter (GUS) gene under the control of the *PDF1.2 *promoter in *Nicotiana benthamiana *leaves, in *Agrobacterium tumefaciens*-mediated transient expression (agroinfiltration) assays. For these assays, we prepared a constitutively active form of CPK proteins which lacks junction and calmodulin-like domains and thus no longer shows Ca^2+ ^dependency. As shown in Figure 8B, the reporter gene activity was drastically increased when co-expressed with HsfB2a as effector. The activation by HsfB2a was further enhanced (6.6- and 1.9-fold) when CPK3 and CPK13 were co-expressed, respectively. However, when CPK3 or CPK13 was co-expressed in the absence of HsfB2a, scarcely any increase of the expression of the reporter gene was observed. Similarly, other CPK substrate transcription factors (ERF1 or CZF1/ZFAR1) were co-expressed as effectors, but neither of them resulted in significant transactivation of the GUS marker gene (data not shown).

### Discussion

Regulation of Ca^2+ ^homeostasis is important, particularly when Ca^2+ ^is involved as a signaling ion. In plant cells, Ca^2+^-binding proteins also serve as regulators of internal free Ca^2+ ^levels. Protein phosphorylation is the most common type of post-translational modification, and functions through phosphorylation-induced conformational changes [24]. Since CPKs may be involved in the specificity and cross-talk of signal transduction for a variety of biotic and abiotic stresses, their possible involvement in active signaling cascades in herbivore responses needs to be investigated [6]. The present study provides a new view of a signaling network for plant-insect interactions. This cascade is not involved in the phytohormone (JA, ethylene and ABA)-related signaling pathways, but rather is able to directly impact transcription factors for defense responses. In fact, we did not observe striking effects of loss of CPK function on the biomass of *S. littoralis *larvae by feeding *cpk3 *or *cpk13 *mutants compared to WT plants for up to 3 days (Additional file 5). This suggests that more genes than just *PDF1.2 *contribute in a complex manner to the onset of acquired resistance to the generalist herbivore *S. littoralis*. For instance, compared to the levels in WT leaves infested by *S. littoralis*, the leaf transcript levels of *Thi2.1 *were higher in *cpk13 *after 24 h but lower after 6 h (Figure 2).

Herbivory responses in Arabidopsis may not be mediated strikingly by the ABA signaling network (Figures 3 and 4). In guard cells, CPK3 appears to act in the phosphorylation of plasma membrane S-type anion channels for the Ca^2+^-reactive stomatal closure response [9]. Our data show that a loss of CPK3 function may consequently lead to an increase of cytosolic Ca^2+ ^concentration in the infested leaf cells (Figure 5). If we consider that ABA activation of plasma membrane Ca^2+^-permeable channels is, in contrast, impaired in *cpk3 *or double *cpk3cpk6 *mutant guard cells [9], we can argue that mesophyll cells, which are the most responsive to herbivore attack, respond in a different way compared to guard cells. In summary, the present findings are consistent with a model in which additional signaling branches function in the herbivory signal transduction network in parallel to CPK3-imposed feedback regulation of Ca^2+ ^channels, and these additional branches are different from those of the stomatal closure response controlled via ABA signaling. In contrast to CPK3, CPK13 does not act upstream of herbivore-stimulated Ca^2+ ^transients (Figure 5).

Screening of the databases for *cis*-acting regulatory DNA elements revealed the presence of a GCC box ((A)GCCGCC [25]) in the *PDF1.2 *promoter that is potentially recognized by ERF1, which is one of the CPK3 substrates (Figure 7) and a potent regulator of *PDF1.2 *[26]. However, our agroinfiltration assays showed that ERF1 as a cofactor failed to transactivate the reporter GUS gene under the control of the GCC consensus sequence (fused to a minimum TATA box) and the *PDF1.2 *promoter in *N. benthamiana *(data not shown). We therefore infer that ERF1 may interact only indirectly with the *PDF1.2 *promoter, similarly to MYC2 [27]. Otherwise, additional *cis*/*trans*-factors or protein modifications might be required to fully facilitate this hetero *planta s*ystem.

According to our agroinfiltration assays, CPK3-or CPK13-derived phosphorylation(s) of a heat shock factor (HsfB2a) appeared to be directly and positively involved in *PDF1.2 *transcriptional regulation (Figure 8B). Heat shock factors (Hsfs) are well known to function in the regulation of stress-inducible genes (e.g., *Hsp*) by recognizing a conserved binding motif (heat shock element [HSE]: three inverted repeats of nGAAn units [28,29]). However, the *PDF1.2 *promoter lacks intact HSE motifs. It is therefore considered that HsfB2a can act as part of the transcriptional machinery of *PDF1.2 *transcription by directly binding to non-HSE sequence(s) present in the promoter, and then CPK3 and CPK13 phosphorylate HsfB2a to modulate its activity. Similarly, Kumar et al. [30] reported that double knockout *hsfb1*/*hsfb2b *plants showed up-regulation of the basal mRNA-levels of *PDF1.2 *in mutant plants. Therefore, it will be of great interest to identify a novel non-HSE DNA recognition site for the class B-Hsf transcription factors.

HsfB2a belongs to the Hsf class B transcription factors (B-Hsfs). However, the function of class B-Hsfs differs from that of class A-Hsfs due to a structural variation within the oligomerization domain and the lack of an AHA-motif, which is required for the transcriptional activation function of class A-Hsfs [31]. Since B-Hsfs have the capacity to bind to similar or the same HSE sites in the heat shock gene promoters as class A-Hsfs, most of them may act as repressors of target gene expression [32,33]. If class B-Hsfs generally antagonistically interact with A-Hsfs by binding (or competing for binding) to the HSE consensus sequence, their regulatory mechanisms would in most cases be different from the positive regulation of the herbivore-induced *PDF1.2 *promoter lacking intact HSE motifs. A-Hsfs should be investigated as possible additional cofactors in further studies.

### Conclusions

These results reveal the involvement of two Arabidopsis CPKs (CPK3 and CPK13) in the herbivory-induced signaling network via HsfB2a-mediated regulation of the defense-related transcriptional machinery. To reveal whether protein phosphorylation has significant effects on the transcript levels in response to feeding by caterpillars, future studies such as genetic analyses investigating double mutants defective for both CPKs and substrates or plants overexpressing those genes will be needed.

## Methods

### Plants, caterpillars, and treatments

Arabidopsis plants (Col-0) were grown in soil. Individual plants were grown in plastic pots in a growth chamber at 22°C (160 *μ*E m^-2 ^s^-1 ^during a 12-h photoperiod) for 5 weeks. Larvae of *S. littoralis *Boisd. (Lepidoptera, Noctuidae) were reared on artificial diet [34] in a plastic box (25 ± 1°C; 14 h light: 10 h dark). For the herbivory treatments, three second-and third-instar larvae were placed on leaves of an *Arabidopsis *plant (non bolting). For chemical treatment, (+)-jasmonic acid or abscisic acid (Sigma-Aldrich) at a concentration of 0.3 mM or 50 μM, respectively, in aqueous solution, or ethephon (0.3 mM, Sigma-Aldrich) at a concentration of 3 mM in sodium phosphate buffer (50 mM, pH7), was evenly sprayed (about 3 ml) onto intact plants.

### Genetic analysis

The homozygous T-DNA lines were screened using two PCR tests http://signal.salk.edu/tdnaprimers.html using either a pair of primers consisting of a T-DNA left border primer (LBa1) and a specific primer for the corresponding T-DNA insertion region or a pair of gene-specific primers which straddle the outer T-DNA flanking regions. Genomic DNAs were isolated from the leaves following the CTAB method [35], and were used as the template for polymerase chain reaction (PCR) following the method described in: http://signal.salk.edu/tdnaprimers.2.html. These PCR analyses were performed at least twice through the two generations along T3 or T4 to confirm whether the lines are certainly homozygous.

### Reverse transcription (RT)-PCR and real-time PCR

Total RNA was isolated from leaf tissues using a Qiagen RNeasy Plant RNA kit and an RNase-Free DNase Set (Qiagen) following the manufacturer's protocol. First-strand cDNA was synthesized using SuperScript II RT, oligo(dT)_12-18 _primer, and 1 μg of total RNA at 42°C for 50 min. The real-time PCR was done on an M×3000 P Real-Time PCR System (Stratagene) [36]. PCR conditions were chosen by comparing threshold values in a dilution series of the RT product, followed by non-RT template control and non-template control for each primer pair. Relative RNA levels were calibrated and normalized with the level of ACT1 (At2G37620) mRNA.

### Quantification of JA, JA-Ile, ABA and ethylene

Leaves (200 mg) were harvested in FastPrep tubes containing 0.9 g of FastPrep matrix (BIO 101, Vista, CA), flash-frozen in liquid nitrogen, and stored at -80°C until use. Ethyl acetate (1 ml), spiked with 200 ng each of internal standards^ 2^H_2_-JA, ^13^C_6_-JA-Ile and ^2^H_6_-ABA, was added to each sample and then the mixture was homogenized using a FastPrep homogenizer (Savant Instruments, Holbrook, NY). After centrifugation at 12,000 *g *for 20 min at 4°C, supernatants were transferred to Eppendorf tubes. Each pellet was re-extracted with 1 ml of ethyl acetate and centrifuged; supernatants were combined and then evaporated to dryness under vacuum. The residue was resuspended in 0.5 ml of 70% methanol/water (v/v) and centrifuged to clarify phases, and the supernatants were analyzed using a 1200 L LC/MS system (Varian, Palo Alto, CA) as described in [37].

Ethylene production was measured in real-time with a photoacoustic laser spectrometer (ETH-PAC1-TR, http://www.invivo-gmbh.de, Germany) in combination with a gas multiplexer (4 channels) [38], in which an Arabidopsis plant in a pot was infested with larvae for up to 24 h.

### Intracellular calcium concentration measurement

A solution of Fluo-3 AM (acetoxy-methyl ester of Fluo-3, 5 μM, Fluka, Buchs, Switzerland), 0.5 mM calcium sulphate, and 2.5 μM DCMU [3-(3',4'-dichlorphenyl)-1,1-dimethylurea] in 50 mM MES buffer, pH 6.0, was used for initial treatment of leaves of an intact Arabidopsis plant as previously described [39]. A leaf was cut once with a razor blade in order to allow the dye to enter the tissues. One hour after treatment with Fluo-3 AM, the leaf was fixed on an Olympus FLUOview confocal laser scanning microscope (CLSM) stage without detaching it from the plant. The microscope was operated with a krypton/argon laser at 488 nm and 568 nm wavelengths: the first wavelength excited the Fluo-3 dye emitting green light, while the second excited mostly chloroplasts emitting red fluorescence. Images generated using FluoView software were analyzed with NIH Image J software. Earlier microscopic analysis showed the false-color subcellular localization of the dyes, which indicated that the dyes are loaded mainly into the cytosol [40].

### Vector construction and transient expression of GFP fusion proteins

Gateway Technology (Invitrogen) was used for the generation of p2GWF7 transformation constructs, which consisted of a target gene (CPK13 ORF cDNA) bearing an N-terminal fusion to eGFP under the control of the dual Cauliflower Mosaic Virus 35S promoter for plant transformations [41]. The attB adaptor-bearing PCR primers (see Additional file 6) were designed for the generation of attB PCR products for recombination with the donor vector pDONRzeo via BP Clonase reactions (Invitrogen). Fully sequenced entry clones were recombined in LR Clonase reactions with the p2GWF7 vector [41]. One microgram of the plasmid was precipitated onto 1.0-μm spherical gold particles (Bio-Rad). Onion peels were bombarded using a particle gun PDS-1000/He (Bio-Rad) according to the manufacturer's instructions. After 24 h, GFP fluorescence of the onion peel was observed under a BX51 fluorescent microscope (OLYMPUS).

### Transcription and cell-free protein synthesis

In order to prepare recombinant proteins fused with GST or a biotin ligase recognition site at their N-terminus, Riken Arabidopsis full-length cDNA clones (RAFL) were used. The DNA constructs were made, according to [42], by two rounds of "Split-Primer" PCR, with the first PCR performed with a target protein-specific primer (5'-CCACCCACCACCACCAatgnnnnnnnnnnnnnnnn-3'; lowercase indicates the 5'-coding region of the target gene) and the AODA2306 primer. The second PCR was performed with an SPu primer, AODA2303 primer, and a deSP6E02bls-S1 primer, which contains a biotin ligase recognition site sequence (for the CPK substrates). For the CPK-GST fusion proteins, a full-length ORF was reinserted into pEU-E01-GST-TEV-MCS vector (Cell-free Sciences, Yokohama, Japan) and used for *in vitro *transcription. *In vitro *transcription, cell-free protein synthesis, and protein purification were performed as described [42,43].

### Auto-phosphorylation reaction of recombinant CPK

Crude GST-tagged recombinant CPK protein (20-40 μg) produced by the dialysis method [44] was precipitated with glutathione Sepharose™ 4B (GE Healthcare). The protein on the Sepharose was washed twice with PBS buffer and then treated with PBS buffer containing 2.5 mM 1,2-*bis*-(2-aminophenoxy)ethane-*N,N,N',N'*-tetra acetic acid (BAPTA, Sigma-Aldrich) at 4°C for 10 min, to remove free Ca^2^. After washing with PBS buffer, the recombinant CPK was eluted with 45 μl of PBS buffer containing 0.1 U of AcTEV protease (Invitrogen), which cleaved the CPK from the GST-tag. Autophosphorylation reactions were carried out in 10 μl of total reaction mixture containing 50 mM Tris-HCl (pH 7.5), 1 μl of partially purified CPK, 0 to 500 μM CaCl_2_, 10 mM potassium acetate, 50 mM MgCl_2_, 0.5 mM DTT and 37 kBq of [γ-^32^P] ATP at 30°C for 30 min. To stop the reaction, 5 μl of 3x-sample buffer [150 mM Tris-HCl (pH 6.8), 6% SDS, 3% 2-mercaptoethanol, and 0.012% bromophenol blue] was added to the reaction mixture. After boiling the reaction mixture, recombinant CPK was separated by 12.5% SDS-polyacrylamide gel electrophoresis (PAGE). The labeled signals were detected with BAS-2500 (FUJIFILM, Japan).

### *In vitro *phosphorylation of target protein

*In vitro *phosphorylation of target was carried out according to a previously described method with a minor modification [44], using partially purified recombinant CPK and substrate. Partially purified GST-tagged recombinant CPK was prepared as described above, but excluding BAPTA from the PBS buffer. Forty microliters of each crude biotinylated substrate protein produced by the bilayer method [43] were attached to Streptavidin Magnesphere Paramagnetics particles (Promega). After washing, the substrate protein on the particles (1-2 μg) was incubated in 15 μl of reaction mixture containing 50 mM Tris-HCl (pH 7.5), 1 μl of purified CPK, 100 μM CaCl_2_, 500 mM potassium acetate, 50 mM MgCl_2_, 0.5 mM DTT and 37 kBq of [γ-^32^P] ATP, in the presence or absence of 500 μM BAPTA at 30°C for 30 min. Following the reaction, the beads were washed twice with PBS, then boiled in sample buffer [50 mM Tris-HCl (pH 6.8), 2% SDS, 1% 2-mercaptoethanol, and 0.004% bromophenol blue]. For the detection method, see above.

### *Agrobacterium tumefaciens-mediated *transient expression (agroinfiltration) in *Nicotiana benthamiana*

A cDNA fragment of a truncated variant lacking junction and calmodulin-like domains was cloned into the pER8 (XVE) binary vector [45]. The full-length coding region of *HsfB2a *was inserted into the GUS reporter gene site of the binary vector pGreen 0229 (35S promoter::GUS including intron) [46]. The 1-kb *PDF1.2 *promoter region upstream of the transcription start site was inserted into the 35S promoter region of the above pGreen-GUS vector.

*Agroinfiltration *was carried out according to a modified protocol from Kobayashi et al. (2007) [47]. Binary plasmids were transformed into *Agrobacterium *strain GV3101, which contains the transformation helper plasmid pSoup [46], and the bacteria were cultured overnight. The culture was diluted 10-fold in Luria-Bertani medium/rifampicin with kanamycin or spectinomycin, and then was cultured until OD_600 _0.6. Cells were harvested by centrifugation and resuspended in 10 mM MES-NaOH, pH 5.6 and 10 mM MgCl_2_. The bacterial suspensions were adjusted to OD_600 _0.5, and then acetosyringone was added to a final concentration of 150 μM. The suspensions were incubated for 2-4 h at 22°C, and a mixture of those carrying CPK, HsfB2a, luciferase (LUC, see below) and *PDF1.2 *promoter::GUS vectors at an approximate ratio (1:1:1:3) was infiltrated into leaves of 4- to 5-week-old *N. benthamiana *plants by using a needleless syringe. One day after bacterial infiltration, β-estradiol (10 μM) was infiltrated into the same position of the leaf to induce the expression of a chimeric transcription activator XVE gene [45], and the plant was kept for 1 day. We then prepared an enzyme extract by homogenization of a leaf with a grinding buffer containing 100 mM potassium phosphate (pH 7.8), 1 mM EDTA, 7 mM 2-mercaptoethanol, 1% Triton X-100 and 10% glycerol, followed by centrifugation. GUS activity was measured by monitoring cleavage of the β-glucuronidase substrate 4-methylumbelliferyl β-D-glucuronide (MUG) [48]. *Agrobacterium *carrying pGreen 0229 (35S promoter::LUC including intron) was used to normalize for the efficiency of agroinfiltration. The luciferase activity in protein extracts was measured using a PicaGene luciferase kit (Toyo Ink, Japan) according to the manufacturer's protocol.

### Data and statistical analysis

At least five repetitions with individual biological sample sets were used for the statistical treatment of the data. The data are expressed as mean values; error bars indicate the standard error. To evaluate the significance of differences of data, ANOVA followed by Fisher's PLSD test was performed.

### Data and statistical analysis

At least five repetitions with individual biological sample sets were used for the statistical treatment of the data. The data are expressed as mean values; error bars indicate the standard error. To evaluate the significance of differences of data, ANOVA followed by Fisher's PLSD test was performed.

## Authors' contributions

CNK carried out all the biological and genetic analyses. HT and AM carried out cell-free protein synthesis and *in vitro *phosphorylation assays and participated in writing the methods section. SQ, CB and SAZ performed the RT-PCR and real-time PCR experiments. MEM and SB carried out calcium measurement. NI and HY helped with agroinfiltration assays and participated in writing the methods section. WB, JT and YE participated in the coordination of the work. TS and GA conceived the intellectual design of the project and wrote the manuscript. All authors read and approved the final manuscript.

## Supplementary Material

Additional file 1 **Molecular analysis of CPK T-DNA insertion mutants and gene expression profiling in the mutants**. A, Disruption of CPK mRNA expression in leaves of the respective *cpk *mutants and the Col-0 wild-type (WT). None of the mutant CPK genes was expressed in the corresponding mutant leaves under the growth conditions, whereas all of them were expressed in WT leaves. B, T-DNA insertion site in *cpk3 *(Salk_022862), *cpk13-1 *(Salk_057893) and *cpk13-2 *(Salk_135795). PCR was performed with a primer pair consisting of a left border primer of the T-DNA and a gene-specific primer, and PCR products were sequenced to determine the T-DNA insertion positions (solid lines). ATG and TGA/TAG indicate start and stop codons. White boxes indicate exons. The T-DNA inserts in *cpk3 *and *cpk13-1 *are located in the first exon, while the insertion in *cpk13-2 *is located in the promoter region upstream of the *CPK13 *gene. Note that Southern blot analyses of homozygous plants showed only a single T-DNA insertion in all four mutants (data not shown). Click here for file

Additional file 2 **Substrate targeting for CPKs**. A table listing protein substrates synthesized using the wheat germ cell-free system. Click here for file

Additional file 3 **Substrate targeting of CPK3 and CPK13**. The quantitative values for data in Figure 7B are shown. Click here for file

Additional file 4 **Supplemental discussion**. Implications for possible involvement of ubiquitination in the CPK signaling pathway are discussed. Click here for file

Additional file 5 **Growth of *S. littoralis *larvae on a WT, *cpk3 *and *cpk13-1 *plant**. Freshly hatched *S. littoralis *larvae were grown on artificial diet. The second instar of the larva was subjected to growth on a WT, *cpk3 *and *cpk13-1 *plant in a pot at 25°C. The larva was allowed to feed for up to 3 days, and its biomass was recorded every 24 h. Click here for file

Additional file 6 **Primers used for this study**. A table listing primers used for this study. Click here for file
