# Supplementary figures and images for: Regulation of Arabidopsis defense responses against Spodoptera littoralis by CPK-mediated calcium signaling
Source: BMC Plant Biol. 2010 May 26;10:97. doi: 10.1186/1471-2229-10-97 (PMC3095362; doi:10.1186/1471-2229-10-97)

## Slide 1
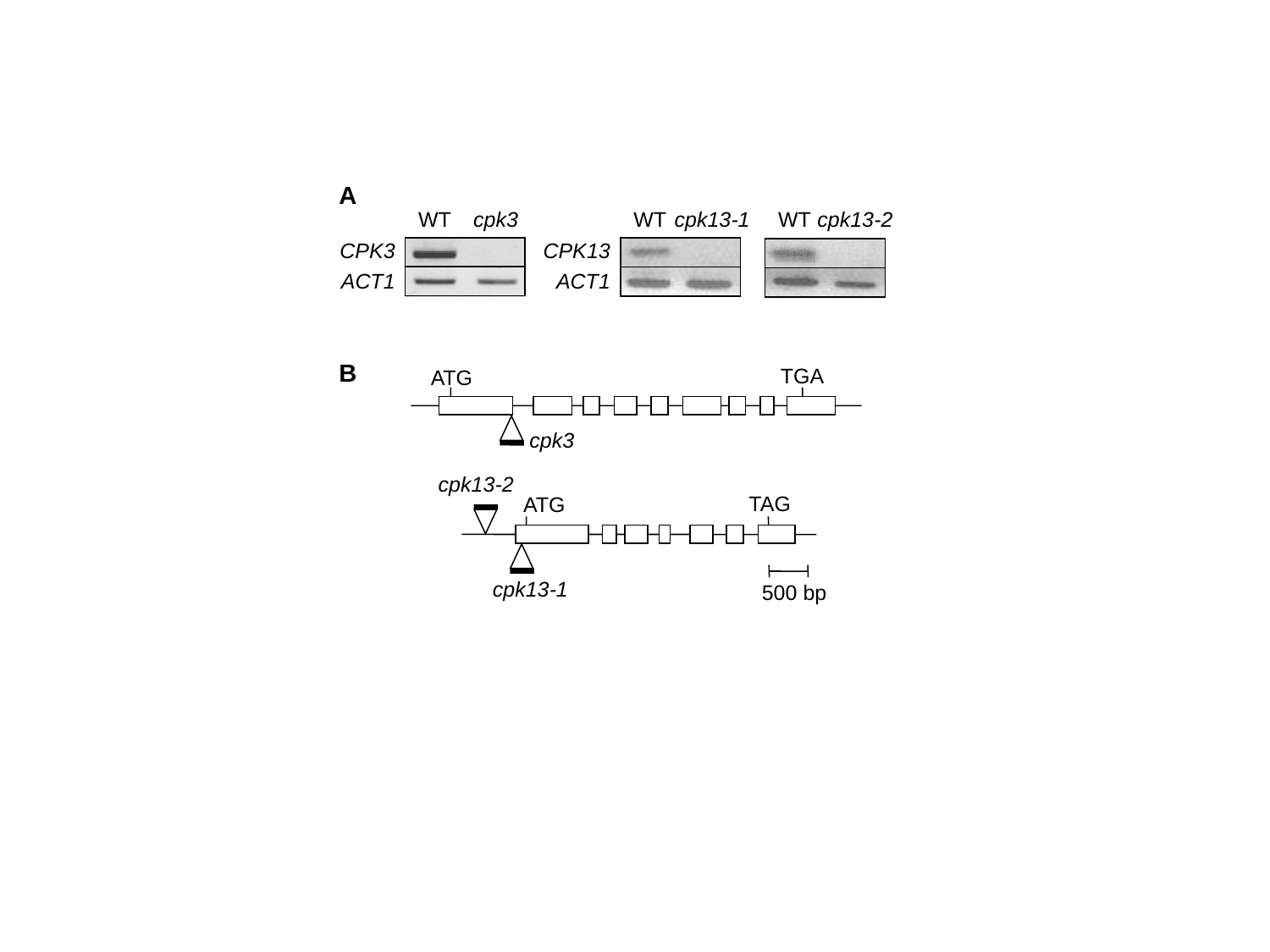

A
WT
cpk3
WT
cpk13-1
WT
cpk13-2
CPK3
CPK13
ACT1
ACT1
B
TGA
ATG
cpk3
cpk13-2
TAG
ATG
cpk13-1
500 bp

Supplement: Additional file 1 — Molecular analysis of CPK T-DNA insertion mutants and gene expression profiling in the mutants. A, Disruption of CPK mRNA expression in leaves of the respective cpk mutants and the Col-0 wild-type (WT). None of the mutant CPK genes was expressed in the corresponding mutant leaves under the growth conditions, whereas all of them were expressed in WT leaves. B, T-DNA insertion site in cpk3 (Salk_022862), cpk13-1 (Salk_057893) and cpk13-2 (Salk_135795). PCR was performed with a primer pair consisting of a left border primer of the T-DNA and a gene-specific primer, and PCR products were sequenced to determine the T-DNA insertion positions (solid lines). ATG and TGA/TAG indicate start and stop codons. White boxes indicate exons. The T-DNA inserts in cpk3 and cpk13-1 are located in the first exon, while the insertion in cpk13-2 is located in the promoter region upstream of the CPK13 gene. Note that Southern blot analyses of homozygous plants showed only a single T-DNA insertion in all four mutants (data not shown). [file 1471-2229-10-97-S1.PPT]

## Slide 1
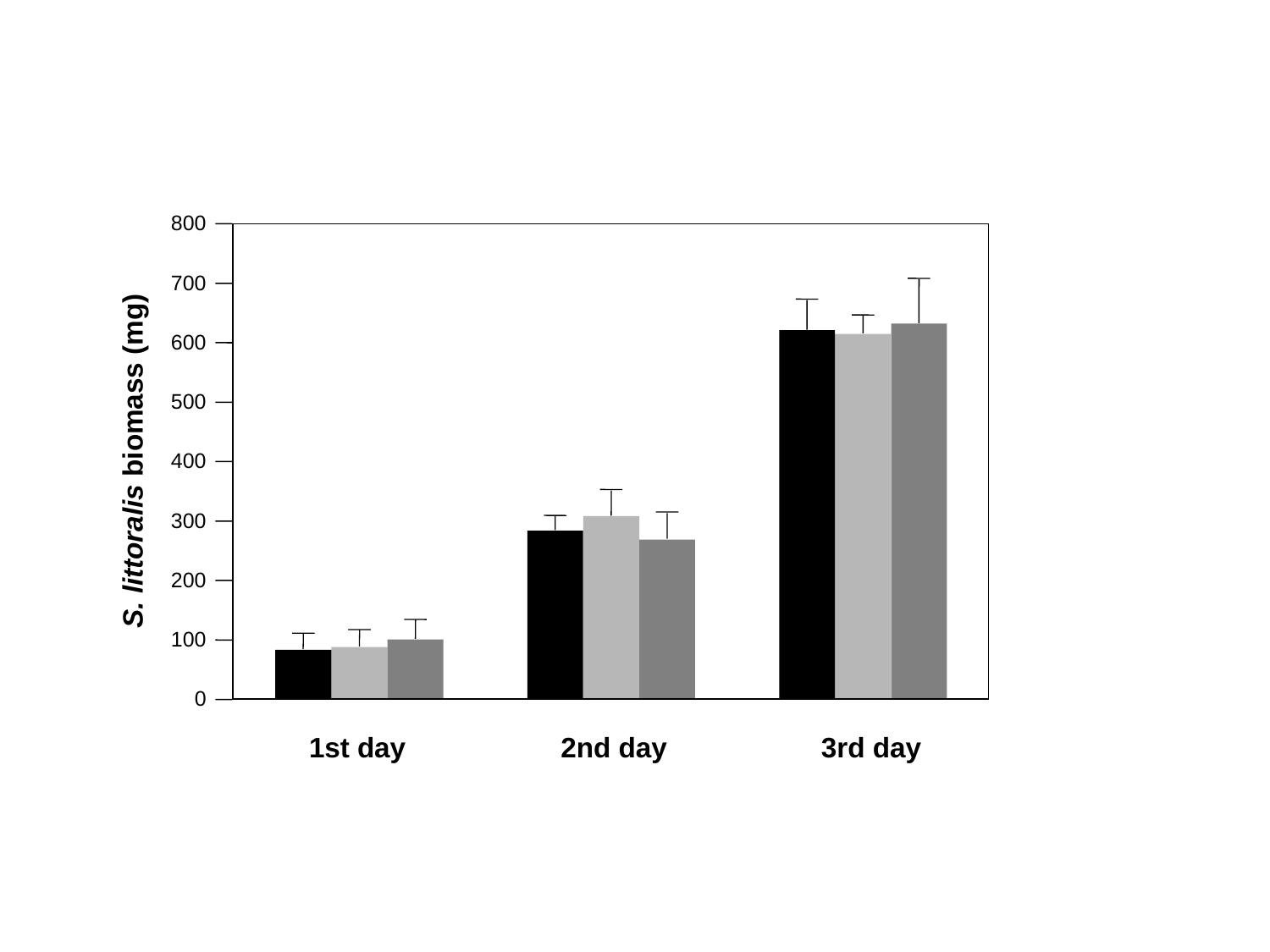

800
700
600
500
 S. littoralis biomass (mg)
400
300
200
100
0
1st day
2nd day
3rd day

Supplement: Additional file 5 — Growth of S. littoralis larvae on a WT, cpk3 and cpk13-1 plant. Freshly hatched S. littoralis larvae were grown on artificial diet. The second instar of the larva was subjected to growth on a WT, cpk3 and cpk13-1 plant in a pot at 25°C. The larva was allowed to feed for up to 3 days, and its biomass was recorded every 24 h. [file 1471-2229-10-97-S5.PPT]
