# Supplementary material for: Regulation of Arabidopsis defense responses against Spodoptera littoralis by CPK-mediated calcium signaling
Source: BMC Plant Biol. 2010 May 26;10:97. doi: 10.1186/1471-2229-10-97 (PMC3095362; doi:10.1186/1471-2229-10-97)
Supplement: Additional file 2 — Substrate targeting for CPKs. A table listing protein substrates synthesized using the wheat germ cell-free system. [file 1471-2229-10-97-S2.DOC]

Substrate targeting for CPKs

| **Annotation** | **AGI code** |
| --- | --- |
| acid-induced protein 7 (IAA7) | At3g23050 |
| AHBP-1B | At5g06950 |
| AP2 domain transcription factor | At2g20880 |
| AP2 domain-containing protein | At5g52020 |
| AtABF4 | At3g19290 |
| ERF1 | At3g23240 |
| AtERF1 | At4g17500 |
| AtERF2 | At5g47220 |
| AtERF3 | At1g50640 |
| AtERF4 | At3g15210 |
| AtERF5 | At5g47230 |
| AtERF6 | At4g17490 |
| AtERF7 | At3g20310 |
| AtERF8 | At1g53170 |
| AtERF11 | At1g28370 |
| AtERF12 | At1g28360 |
| AtERF13 | At2g44840 |
| AtERF15 | At2g31230 |
| EREBP homolog | At4g34410 |
| AtHsf2 | At1g46264 |
| AtHsf4 | At2g26150 |
| AtHsf5 | At2g41690 |
| AtHsf6 | At3g02990 |
| AtHsf7 | At3g22830 |
| AtHsf14 | At4g18870 |
| AtHsf15 | At4g18880 |
| AtHsf16 | At4g36990 |
| AtHsf18 | At5g16820 |
| AtHsf19 | At5g43840 |
| AtHsf20 | At5g45710 |
| AtHsf21 | At5g54070 |
| AtHsf22 (HsfB2a) | At5g62020 |
| auxin:hydrogen symporter (PIN7) | At1g23080 |
| auxin-induced protein (IAA20) | At2g46990 |
| ethylene insensitive root 1 (EIR1) | At5g57090 |
| cytokinin response factor 5 (CRF5) | At2g46310 |
| bHLH family protein | At2g22770 |
| bZIP transcription factor ATB2 (GBF6) | At4g34590 |
| C2H2-type zinc-finger protein related (FZF) | At2g24500 |
| late elongated hypocotyl (LHY) | At1g01060 |
| CONSTANS B-box zinc-finger family protein | At3g21150 |
| DRE-binding protein (DREB2A) | At5g05410 |
| DRE-binding protein (DRE2B) | At2g38340 |
| homeoboxr protein (ATHB-1) | At3g01470 |
| homeobox protein (ATHB-7) | At2g46680 |
| homeobox protein (ATHB-12) | At3g61890 |
| dlongated hypocotyl 5 (HY5) | At5g11260 |
| MYB3 | At1g22640 |
| MYB4 | At4g38620 |
| MYB7 | At2g16720 |
| MYB15 | At3g23250 |
| MYB34 | At5g60890 |
| MYB44 | At5g67300 |
| MYB47 | At1g18710 |
| MYB70 | At2g23290 |
| MYB73 | At4g37260 |
| MYB75 | At1g56650 |
| MYB96 | At5g62470 |
| MYB family transcription factor | At1g58220 |
| NAC-domain TF | At1g52890 |
| MYC2 | At1g32640 |
| MYC4 | At4g17880 |
| ORA59 | At1g06160 |
| RAP2.4 | At1g78080 |
| RAP2.6 | At1g43160 |
| RAP2.7/TOE1 | At2g28550 |
| RAP2.10 | At4g36900 |
| RAV2 | At1g68840 |
| TGA1 | At5g65210 |
| WRKY6 | At1g62300 |
| WRKY11 | At4g31550 |
| WRKY15 | At2g23320 |
| WRKY17 | At2g24570 |
| WRKY22 | At4g01250 |
| WRKY28 | At4g18170 |
| WRKY30 | At5g24110 |
| WRKY33 | At2g38470 |
| WRKY46 | At2g46400 |
| WRKY53 | At4g23810 |
| WRKY60 | At2g25000 |
| WRKY70 | At3g56400 |
| zinc finger (AN1-like) family protein | At3g52800 |
| znc-finger protein (PMZ) | At3g28210 |
| zinc-finger protein (Zat12) | At5g59820 |
| zinc finger (AN1-like) family protein | At4g12040 |
| zinc finger (AN1-like) family protein | At2g27580 |
| zinc finger (GATA type) family protein | At4g36240 |
| C2H2 zinc finger transcription factor | At5g04340 |
| CZF1/ZFAR1 | At2g40140 |
| DOF zinc finger protein | At2g28510 |
| DOF zinc finger protein | At2g34140 |
| RING-H2 finger protein (RHA3b) | At4g35480 |
| C3HC4-type RING finger family protein | At5g42940 |
| C3HC4-type RING finger family protein | At5g27420 |
| phytochrome interacting factor 4 (PIF4) | At2g43010 |
| bZIP transcription factor family protein | At3g51960 |
| bZIP transcription factor family protein | At2g40620 |
| salt-tolerance zinc-finger protein (ZAT10) | At1g27730 |
| salt tolerance during germination 1 (TAFII15) | At4g31720 |
| ATL2 | At3g16720 |
