# Supplementary material for: Regulation of Arabidopsis defense responses against Spodoptera littoralis by CPK-mediated calcium signaling
Source: BMC Plant Biol. 2010 May 26;10:97. doi: 10.1186/1471-2229-10-97 (PMC3095362; doi:10.1186/1471-2229-10-97)
Supplement: Additional file 3 — Substrate targeting of CPK3 and CPK13. The quantitative values for data in Figure 7B are shown. [file 1471-2229-10-97-S3.PPT]

## Slide 1
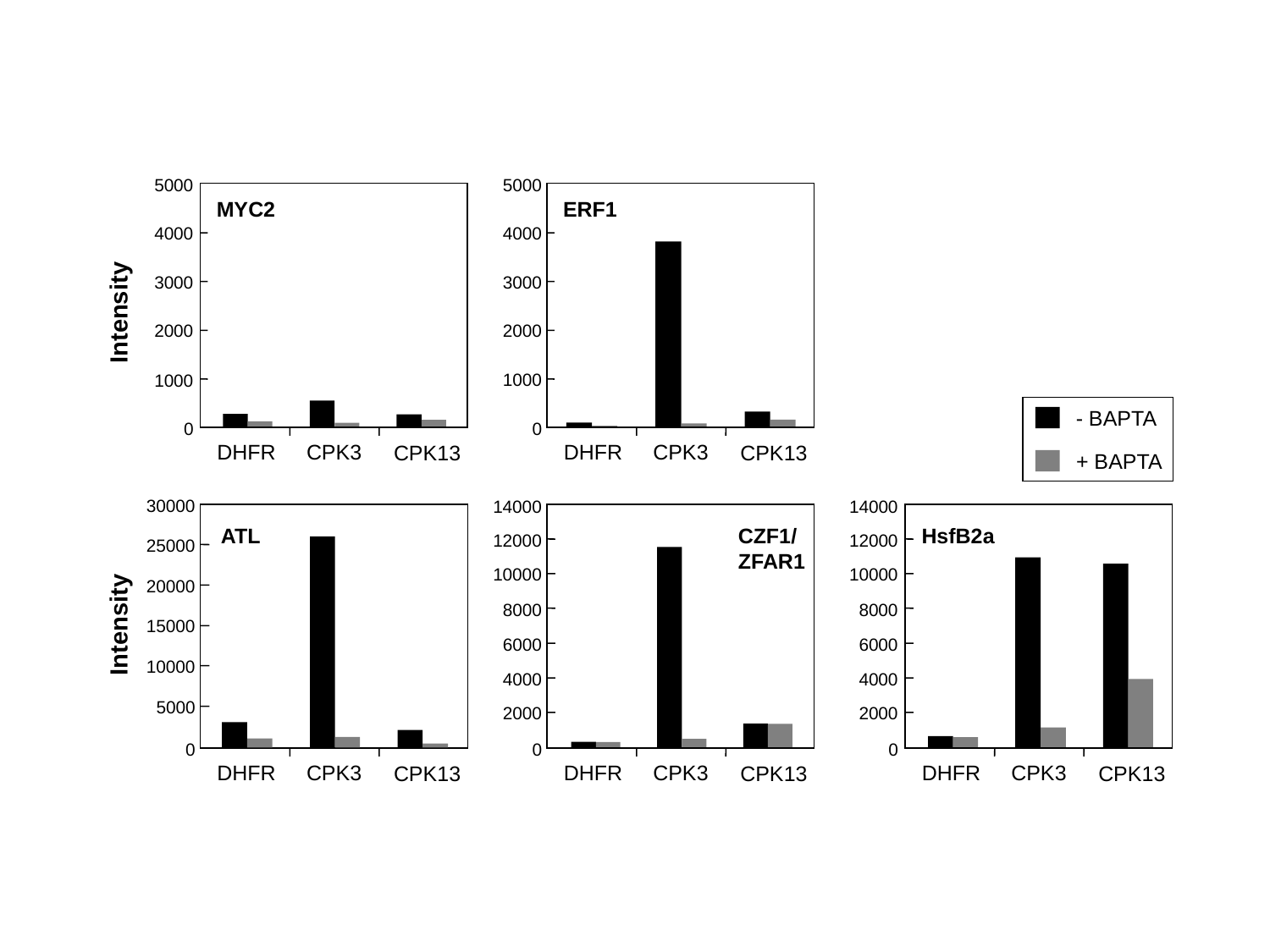

5000
MYC2
4000
3000
2000
1000
0
DHFR
CPK3
CPK13
5000
ERF1
4000
3000
2000
1000
0
DHFR
CPK3
CPK13
Intensity
- BAPTA
+ BAPTA
30000
ATL
25000
20000
15000
10000
5000
0
DHFR
CPK3
CPK13
14000
CZF1/
ZFAR1
12000
10000
8000
6000
4000
2000
0
DHFR
CPK3
CPK13
14000
HsfB2a
12000
10000
8000
6000
4000
2000
0
DHFR
CPK3
CPK13
Intensity
