# Supplementary material for: Regulation of Arabidopsis defense responses against Spodoptera littoralis by CPK-mediated calcium signaling
Source: BMC Plant Biol. 2010 May 26;10:97. doi: 10.1186/1471-2229-10-97 (PMC3095362; doi:10.1186/1471-2229-10-97)
Supplement: Additional file 4 — Supplemental discussion. Implications for possible involvement of ubiquitination in the CPK signaling pathway are discussed. [file 1471-2229-10-97-S4.DOC]

Ubiquitination is common system of post-translational modification and controls protein stability or protein-protein interactions [1]. Our results indicate that three classes of enzymes (E1, E2 and E2 ligases) are needed to participate in an enzymatic cascade (data not shown) and thus imply that ATL2 may be required to specify the substrate specificity and to mediate the transfer of the ubiquitin, as it is for other E3 ubiquitin ligases [2]. Recent findings by Chini et al. (2007) and Thines et al. (2007) led to the proposal that the ubiquitination of JAZ1 and JAI3/JAZ3 with COI1 (an F-box protein) is required for the activation of JA-responsive gene (e.g., *PDF1.2*) expression [3, 4], implying that ubiquitination is an essential process for facilitating and modulating the signal transduction for herbivore defense. Furthermore, a total of 30 ubiquitin-ligase genes were reported to be responsive to chitin, which is found in the cell walls of true fungi and the exoskeleton of insects and nematodes [5].

Since ATL2 is a member of the RING-type E3 ligases, its ubiquitination machinery may be different from that of SCF^COI1^ and thus their substrate targeting might be distinct. It has been documented very recently that screening of ATL2-interacting proteins using a yeast two-hybrid screen mined several transcription factors (including bHLH, NAC and G2-like family) as potential targets of ATL2 (http://abstracts.aspb.org/pb2009/public/P48/P48076.html). Furthermore, direct phosphorylation of E3 ubiquitin ligase was recently reported to induce a conformational change that greatly enhances the catalytic activity of an E3 ligase in T cells [6].

**References**

1. Liu YC: **Ubiquitin ligases and the immune response**. *Annu Rev Immunol* 2004, **22**:81-127.

2. Glickman MH, Ciechanover A: **The ubiquitin-proteasome proteolytic pathway: destruction for the sake of construction**. *Physiol Rev* 2002, **82**:373-428.

3. Chini A, Fonseca S, Fernandez G, Adie B, Chico JM, Lorenzo O, Garcia-Casado G, Lopez-Vidriero I, Lozano FM, Ponce MR *et al*: **The JAZ family of repressors is the missing link in jasmonate signalling**. *Nature* 2007, **448**:666-671.

4. Thines B, Katsir L, Melotto M, Niu Y, Mandaokar A, Liu G, Nomura K, He SY, Howe GA, Browse J: **JAZ repressor proteins are targets of the SCF(COI1) complex during jasmonate signalling**. *Nature* 2007, **448**:661-665.

5. Libault M, Wan J, Czechowski T, Udvardi M, Stacey G: **Identification of 118 *Arabidopsis* transcription factor and 30 ubiquitin-ligase genes responding to chitin, a plant-defense elicitor**. *Mol Plant Microbe Interact* 2007, **20**:900-911.

6. Gallagher E, Gao M, Liu YC, Karin M: **Activation of the E3 ubiquitin ligase Itch through a phosphorylation-induced conformational change**. *Proc Natl Acad Sci U S A* 2006, **103**:1717-1722.
