# Supplementary material for: Regulation of Arabidopsis defense responses against Spodoptera littoralis by CPK-mediated calcium signaling
Source: BMC Plant Biol. 2010 May 26;10:97. doi: 10.1186/1471-2229-10-97 (PMC3095362; doi:10.1186/1471-2229-10-97)
Supplement: Additional file 6 — Primers used for this study. A table listing primers used for this study. [file 1471-2229-10-97-S6.DOC]

#### Primers used for this study

**Gene Purpose Sequence (5’ to 3’)**

LBa1 Genome PCR TGGTTCACGTAGTGGGCCATCG

*CPK3* Genome PCR (F) TTCTGGGGCAACATAGTATGC

(Salk_022862) Genome PCR (R) GTCCTCCTCCTCCTCTTCC

*CPK13* Genome PCR (F) TGACATTTCAAAATCTTAAATGGC

(Salk_057893) Genome PCR (R) GATCGATTTGCAAGCGAGTAG

*CPK13* Genome PCR (F) CAAAGCTCCATAACCAAATGC

(Salk_135795) Genome PCR (R) CATTGTCATAGTTGTTCAGGTGG

*HsfB2a* Genome PCR (F) TGCAATGAGCAAAACAGTGAG

(Salk_027578) Genome PCR (R) ACGGAGAAGACGTTTTTCTCC

*CPK3* RT-PCR (F) TGTTAAACCTGCCGGAGAAC

(At4g23650) RT-PCR (R) TCCCCTCCTTCACACAACTC

*CPK13* RT-PCR (F) tgtatcgaggtcctcgcgt

(At3g51850) RT-PCR (R) ccgacgctttggatcaggctctaac

*ACT1* RT-PCR (F) GAGACAGCCAAAACCAGCTC

(At2G37620) RT-PCR (R) TGAACAATCGATGGACCTGA

*ACT1* Real-time PCR (F) TGCACTTCCACATGCTATCC

Real-time PCR (R) GAGCTGGTTTTGGCTGTCTC

*PDF1.2* Real-time PCR (F) CATGGCTAAGTTTGCTTCCA

(At5g44420) Real-time PCR (R) GTTGCATGATCCATGTTTGG

*Thi2.1* Real-time PCR (F) GTTGGGTAAACGCCATTCTC

(At1g72260) Real-time PCR (R) CCCAGGTGGGACTACATAGC

*VSP2* Real-time PCR (F) CCGTTGGAAGTTGTGGAAGAAT

(At5g24770) Real-time PCR (R) TCTTCACGAGACTCTTCCTC

*CPK13* p2GWF7 attb (F) ggggacaagtttgtacaaaaaagcaggc

ttcaccatgggaaactgttgcagatctcc

p2GWF7 attb (R) ggggaccactttgtacaagaaagctg

ggtgttcgttgcctaggttca

*CPK3* pEU-GST-TEV-MCS (F) GAGACTCGAGATGGGCCACAGACACAGCA

pEU-GST-TEV-MCS (R) GAGAACTAGTTCACATTCTGCGTCGGTTTG

*CPK13* pEU-GST-TEV-MCS (F) GAGACTCGAGATGGGAAACTGTTGCAGATC

pEU-GST-TEV-MCS (R) GAGAACTAGTCTATTCGTTGCCTAGGTTCA

*CPK3* pRE8(XVE) (F) GAGACTCGAGATGGGCCACAGACACAGCAAG

pRE8(XVE) (R) GAGAACTAGTCTAAATCCACGGATGATTTAGCAC

*CPK13* pRE8(XVE) (F) GAGACTCGAGATGGGAAACTGTTGCAGATCTCC

pRE8(XVE) (R) GAGAACTAGTCTAAGCACTTGCTTTGCAGTCAGC

*ERF1* pGreen0229 (F) GAGAATCGATATGGATCCATTTTTAATTC

pGreen0229 (R) GAGAACTAGTCCAAGTCCCACTATTTTC

*HsfB2a* pGreen0229 (F) GAGATTCGAAATGAATTCGCCGCCGGTT

pGreen0229 (R) GAGAGGATCCATTACAAACTCTCTGATT

*CZF1/ZFAR1* pGreen0229 (F) GAGAATCGATATGTGCGGTGCAAAGAGC

pGreen0229 (R) GAGAGGATCCTGCCACAATCTGCTGCTCATG

*PDF1.2* promoter pGreen0229 (F) GAGAGAATTCCCGAGGTGCATCGTTTCTAC

pGreen0229 (R) GAGAATCGATGAAGAAATAAGCCAAAGATCA

*MYC2* “Split-Primer” PCR (F) CCACCCACCACCACCAATGACTGATTACCGGCTAC

*ERF1* “Split-Primer” PCR (F) CCACCCACCACCACCAATGGATCCATTTTTAATTC

*HsfB2a* “Split-Primer” PCR (F) CCACCCACCACCACCAATGATTACCGGAGAATCA

*CZF1/ZFAR1* “Split-Primer” PCR (F) CCACCCACCACCACCAATGTGCGGTGCAAAGAGC

*ATL2* “Split-Primer” PCR (F) CCACCCACCACCACCAATGAACTCCAACGACCAG

*AtUBC8* “Split-Primer” PCR (F) CCACCCACCACCACCAATGGCTTCGAAACGGATCTT

SPu “Split-Primer” PCR (F) GCGTAGCATTTAGGTGACACT

AODA2306 “Split-Primer” PCR (R) AGCGTCAGACCCCGTAGAAA

AODA2303 “Split-Primer” PCR (R) GTCAGACCCCGTAGAAAAGA

deSP6E02bls-S1 “Split-Primer” PCR (F) GGTGACACTATAGAACTCACCTATCTCTCTACACAA

AACATTTCCCTACATACAACTTTCAACTTCCTATTAT

GGGCCTGAACGACATCTTCGAGGCCCAGAAGATCG

AGTGGCACGAACTCCACCCACCACCACCAATG

(F) and (R) indicate the forward and reverse primers, respectively.
